# Relative Expression of Vitamin D Hydroxylases, *CYP27B1* and *CYP24A1*, and of Cyclooxygenase-2 and Heterogeneity of Human Colorectal Cancer in Relation to Age, Gender, Tumor Location, and Malignancy: Results from Factor and Cluster Analysis

**DOI:** 10.3390/cancers4030763

**Published:** 2012-07-26

**Authors:** Wolfgang Brozek, Teresa Manhardt, Enikö Kállay, Meinrad Peterlik, Heide S. Cross

**Affiliations:** Department of Pathophysiology, Medical University of Vienna, Waehringer Guertel 18-20, A-1090 Vienna, Austria; E-Mails: teresa.manhardt@meduniwien.ac.at (T.M.); enikoe.kallay@meduniwien.ac.at (E.K.); meinrad.peterlik@meduniwien.ac.at (M.P.); heide.cross@meduniwien.ac.at (H.S.C.)

**Keywords:** colon cancer, inflammation, grading, tumor progression, modeling, 1,25-dihydroxyvitamin D_3_, 25-hydroxyvitamin D-24-hydroxylase, 25-hydroxyvitamin D-1α-hydroxylase, vitamin D receptor

## Abstract

Previous studies on the significance of vitamin D insufficiency and chronic inflammation in colorectal cancer development clearly indicated that maintenance of cellular homeostasis in the large intestinal epithelium requires balanced interaction of 1,25-(OH)_2_D_3_ and prostaglandin cellular signaling networks. The present study addresses the question how colorectal cancer pathogenesis depends on alterations of activities of vitamin D hydroxylases, *i.e.*, *CYP27B1*-encoded 25-hydroxyvitamin D-1α-hydroxylase and *CYP24A1*-encoded 25-hydroxyvitamin D-24-hydroxylase, and inflammation-induced cyclooxygenase-2 (COX-2). Data from 105 cancer patients on *CYP27B1*, *VDR*, *CYP24A1*, and *COX-2* mRNA expression in relation to tumor grade, anatomical location, gender and age were fit into a multivariate model of exploratory factor analysis. Nearly identical results were obtained by the principal factor and the maximum likelihood method, and these were confirmed by hierarchical cluster analysis: Within the eight mutually dependent variables studied four independent constellations were found that identify different features of colorectal cancer pathogenesis: (i) Escape of COX-2 activity from restraints by the *CYP27B1/VDR* system can initiate cancer growth anywhere in the colorectum regardless of age and gender; (ii) variations in *COX-2* expression are mainly responsible for differences in cancer incidence in relation to tumor location; (iii) advancing age has a strong gender-specific influence on cancer incidence; (iv) progression from well differentiated to undifferentiated cancer is solely associated with a rise in *CYP24A1* expression.

## 1. Introduction

**Figure 1:**
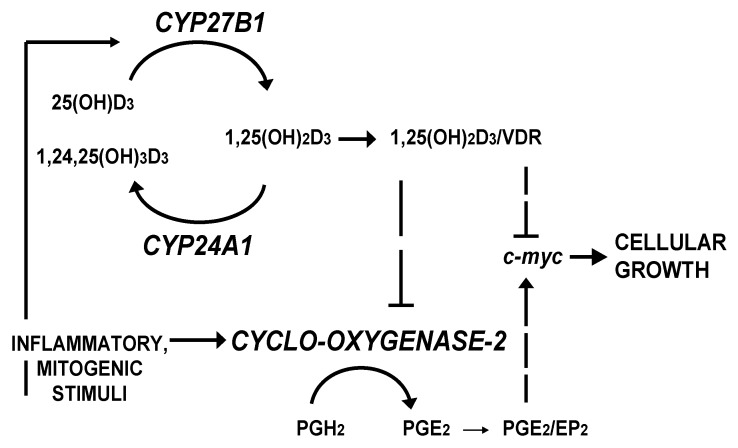
Interaction between the vitamin D endocrine system and the inflammatory signaling pathway in control of cancer cell growth (for details, see [4,5]).

Colorectal carcinomas display a high degree of heterogeneity as incidence, malignancy and clinical outcome differ widely in relation to age, gender and tumor location in the colorectum [1,2]. We had raised the question whether variations in disease susceptibility reflect changes in the activities of intrinsic systems that inhibit or promote cancer cell growth. There is suggestive evidence of this with respect to the components of the autocrine/paracrine vitamin D system of the gut, which produces the potent anti-mitogen 1,25-dihydroxyvitamin D_3_ [1,25-(OH)2D3], and of inflammation-induced cyclooxygenase-2 (COX-2), which catalyzes the conversion of arachidonic acid to growth stimulatory prostaglandin E_2_. Previous studies of the significance of vitamin D insufficiency and chronic inflammation on colorectal cancer development clearly indicated that maintenance of cellular homeostasis in the large intestinal epithelium requires balanced interaction of 1,25-(OH)_2_D_3_ and prostaglandin cellular signaling networks (e.g., [3]) as illustrated in Figure 1.

The hormonally active vitamin D compound, 1,25-(OH)_2_D_3_, when bound to the high-affinity vitamin D receptor (VDR), functions as a potent anti-mitogen in many normal and neoplastic cells [6]. Endogenous synthesis of 1,25-(OH)_2_D_3_ from 25-hydroxyvitamin D (25-(OH)D) in the gut mucosa plays an important role in control of cellular growth and differentiation in the colorectal mucosa [5]. A compromised vitamin D status, characterized by low serum levels of 25-(OH)D, may increase the risk of colorectal cancer due to limited availability of 25-(OH)D as substrate for the colonic *CYP27B1*-encoded 25-hydroxyvitamin D-1α-hydroxylase. Hence, activity of the enzyme may not be high enough to achieve steady-state concentrations of 1,25-(OH)_2_D_3_ in the gut mucosa, which are necessary for efficient control of cell growth. In addition, increased activity of the vitamin D catabolic enzyme, the *CYP24A1-*encoded 25-hydroxyvitamin D-24-hydroxylase, may also curtail anti-proliferative actions of endogenously produced 1,25-(OH)_2_D_3_ [7]. *CYP24A1* has therefore been considered to function as an oncogene for instance in the colon [8].

Anti-inflammatory properties of vitamin D include the ability to down-regulate expression of inflammation-associated *PTGS2*-encoded cyclooxygenase-2 (COX-2) [4,9]. Vitamin D insufficiency may therefore contribute to the persistence of chronic inflammation in the large intestine, which plays an important role in the development not only of colitis-associated but also of sporadic colorectal cancer [10,11]: Increased COX-2 activity enhances the risk of neoplastic transformation in ulcerative colitis by inducing pro-oncogenic mutations in colonic crypts that may give rise to dysplastic and cancerous lesions [12]. In addition, overexpression of *COX-2* accelerates growth of adenomas [13], promotes tumor progression through the adenoma/carcinoma sequence [10,14] and appears to be related to poor survival in colon cancer patients [14]. Both primary and secondary prevention with COX inhibitors lead to decreased incidence of colorectal carcinoma (see e.g., [15]).

In the present study we performed a patient level analysis of *CYP27B1*, *VDR*, *CYP24A1* and *COX-2* mRNA expression in colorectal carcinomas. We were able to demonstrate for the first time by a statistical method also applied in systems biology that variations in the expression of the growth inhibiting *CYP27B1/VDR* system relative to tumorigenic *COX-2* and *CYP24A1* could account for the significant age- and gender-related influence on incidence and malignity of cancers at different anatomical locations within the colorectum, which we had reported previously [2].

## 2. Patients and Methods

### 2.1. Patient Data and Analysis of Tissue Samples

Medical records and surgical biopsies of 105 patients undergoing primary curative surgery at the Hospital Rudolfstiftung, Vienna, were made available to us. Data were pooled according to anatomical subsites, namely proximal or right colon (cecum, ascending and transverse colon), distal or left colon (descending and sigmoid), and rectum. Of the 105 carcinomas, 37 (35.24%) originated in the proximal colon, 31 (29.52%) in the distal colon and 37 (35.24%) in the rectum. Adenocarcinomas were graded according to the WHO classification [16] as well and moderately differentiated, *i.e.*, low-grade (G1 and G2: 59 patients), and poorly and undifferentiated, *i.e.*, high-grade (G3 and G4: 46 patients) cancer. The cohort included 41 male and 64 female patients. Age of patients ranged from 25 to 91 years (median 71) and was similar in female and male patients.

### 2.2. Expression Analysis

Surgical biopsies were analyzed by semi-quantitative RT-PCR for expression of *VDR*, *CYP27B1*, *CYP24A1* and *COX-2* as described before [7,9]. We quantified the RNA and used the same amount of cDNA for amplification. PCR products were separated by electrophoresis on a 2% agarose gel and quantified densitometrically using a video camera imaging system under UV light (Herolab, Wiesloch, Germany). The 500 kb band of the molecular ladder was used as internal control for densitometric evaluation in each gel.

### 2.3. Statistical Methods

RT-PCR data as well as tumor- and patient-related data were statistically evaluated using software packages S-PLUS (Lucent Technologies Inc., Murray Hill, NJ, USA), SPSS (SPSS Inc., Chicago, IL, USA), and NTSYSpc (Applied Biostatistics Inc., Port Jefferson, NY, USA). Testing for normal distribution was carried out with the one-sample Kolmogorov-Smirnov goodness of fit-test for continuous variables, or by the one-sample χ^2^ goodness of fit-test for discrete variables. Continuous, normally distributed variables were subjected to examination with Student’s *t*-test and to correlation analysis with Pearson’s correlation coefficient, whereas discrete ordinal variables as well as continuous variables that displayed no normal distribution were examined with the Wilcoxon rank-sum test and the rank correlation test that employs Spearman’s rho coefficient. Applying a confidence level of 0.95 throughout, differences and correlations were considered statistically significant at *p* < 0.05.

For the multivariate factor analytical model, continuous variables were transformed to normality or near normality employing power transformation techniques and were subsequently standardized, before obtaining the correlation matrix. A scree plot, *i.e.*, a diagram of ranked eigenvalues extracted from the correlation matrix of all variables, suggested a model of four factors due to a marked decline between the 4th and the 5th dimension. Maximum likelihood and principal factor approaches, two commonly applied methods for estimation of parameters of a model, *i.e.*, factor loadings (*cf.* [17]), were used for generation of the initial factor solution, which, for ease of interpretation, was rotated applying the varimax-normalized method. According to Gorsuch [18] only factor loadings of >|0.3| (absolute value) in the reference structure matrix were regarded as salient and were therefore considered for interpretation of factors. Cluster analysis was conducted on the average taxonomic distance matrix of normality-transformed raw data with the unweighted pair-group method, arithmetic average (UPGMA) [19].

## 3. Results

### 3.1. RT-PCR Analysis of mRNA Expression

**Figure 2:**
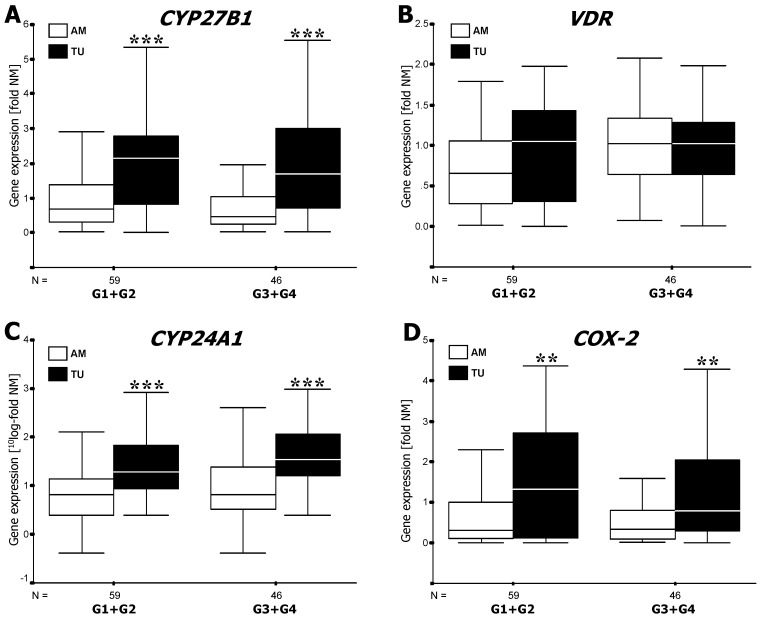
mRNA expression of *CYP27B1* (**A**), *VDR* (**B**), *CYP24A1* (**C**)and *COX-2* (**D**) in cancerous colorectal mucosa (TU) at different stages of malignancy (G1–G4) and in paired tumor-adjacent mucosa (AM). Results of semi-quantitative PCR (see Methods) are shown as boxplots of medians from *n* patients per group. Expression levels in normal mucosa of non-cancer patients were set to 1 for external reference (see [2]). Note that data in Figure 2C are shown on a logarithmic scale. Asterisks indicate statistically significant difference at **, *p* < 0.01; ***, *p* < 0.001.

mRNA expression levels of *CYP27B1*, *CYP24A1* and *COX-2* but not of the VDR were significantly higher in cancerous lesions compared to adjacent mucosa (Figure 2), but varied in different directions during progression from low to high grade malignancy: Compared to the adjacent mucosa, *CYP27B1* expression was four times higher (*p* < 0.001) in both low and high grade cancers (Figure 2A). *COX-2* gene activity in low grade tumors was approximately four times higher (*p* < 0.01) than in tissue outside the tumor area (Figure 2D). High grade cancers expressed still twice as much *COX-2* than the adjacent mucosa (*p* < 0.01) (Figure 2D). Notably, *CYP24A1* mRNA in low grade and high grade lesions was approximately 20, respectively 35 times higher (*p* < 0.001) than in paired specimens of tumor-adjacent mucosa (Figure 2C) (see also [20]). *VDR* mRNA in cancerous lesions was independent of histopathological grading (Figure 2B).

**Figure 3:**
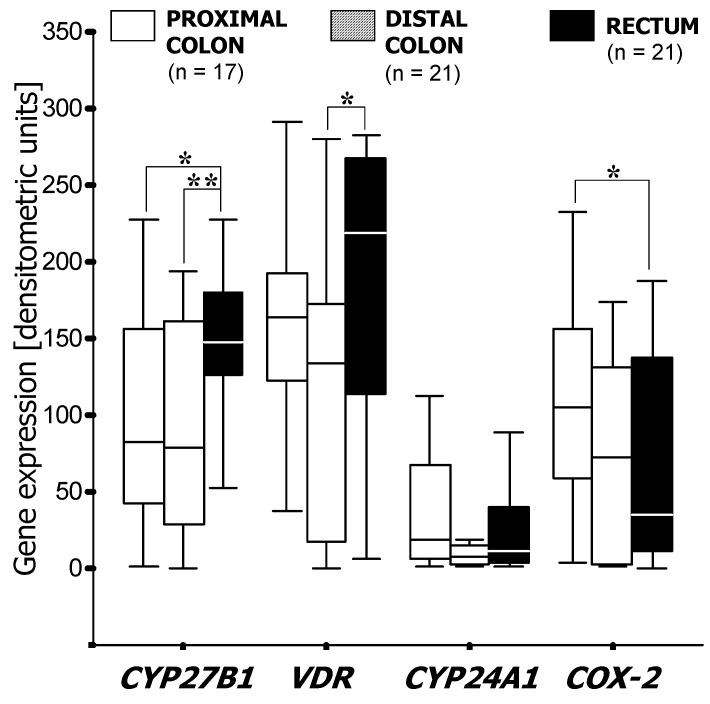
mRNA expression of *VDR*, *CYP27B1*, *CYP24A* and *COX-2* in low grade cancerous lesions at different locations in the colorectum. Results of semi-quantitative PCR (see Methods) are shown as boxplots of medians (from *n* patients per group). Asterisks indicate statistical significance (*, *p* < 0.05; **, *p* < 0.01).

**Figure 4:**
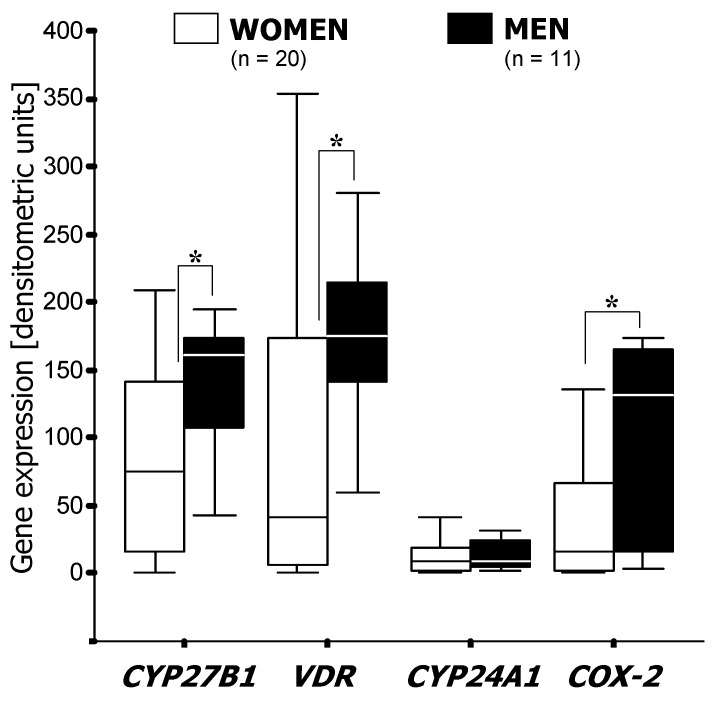
Gender-specific expression of *VDR*, *CYP27B1*, *CYP24A1* and *COX-*2 mRNA in cancerous lesions in distal colon. Results of semi-quantitative PCR (see Methods) are shown as boxplots of medians (from *n* patients per group). Asterisk indicates statistical significance (*, *p* < 0.05).

Subsite-related differences in *CYP27B1* expression were apparent to some extent in low-grade (G1 and G2) cancerous lesions as rectal cancers expressed almost twice as much *CYP27B1* than tumors located in the proximal (*p* < 0.05) and distal colon (*p* < 0.01) (Figure 3). Sub-group analysis according to gender revealed a significant difference in *CYP27B1* mRNA levels between rectal and colon cancers in women (*p* < 0.01) but not in men (not shown). In tumors located in the distal colon, *CYP27B1* was expressed more than twice as much in men than in women (*p* < 0.05) (Figure 4). *COX-2* was elevated in proximal tumors compared with distal and rectal ones (Figure 3). Furthermore, tumors in the distal colon expressed about 10 times more *COX-2* in men than in women (*p* < 0.05) (Figure 4). Transcriptional activity of the *CYP24A1* gene was independent of gender at all sites along the colorectum (data not shown).

### 3.2. Factor and Cluster Analysis

**Figure 5:**
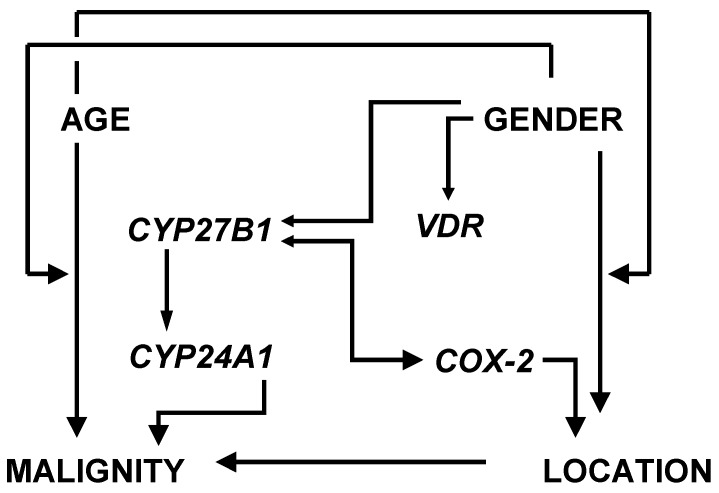
Patient level analysis of mutual correlations between malignancy, age, gender, subsite location, *CYP27B1*, *CYP24A1*, *VDR* and *COX-2* (see also [2]).

The eight variables under investigation, namely malignancy, age, gender, subsite, *CYP27B1*, *CYP24A1*, *VDR* and *COX-2*, are correlated with each other in a complex manner (Figure 5). Therefore, the respective data from patient level analysis were subjected to exploratory factor analysis, a statistical method that allows identification of a small number of independent “factors” in a group of multiple mutually correlated variables. Factors are latent and therefore unobservable common variables that influence more than one measured variable. In our case, a multivariate factor analytical model with a best fit of four latent factors was established (see *2.3. Statistical Methods*). Associations of measured variables with Factors 1*_TU_*–4*_TU_* in colorectal cancer tissue, and with Factors 1*_AM_*–4*_AM_* in adjacent mucosal tissue are listed in Table 1.

**Table 1:** Results from exploratory factor analysis of eight mutually correlated variables with respect to colorectal cancer and adjacent mucosal tissue. Loadings on Factors (F) (=relation between independent factors and measured variables) were calculated by Principal Factor Method (PF) and Maximum Likelihood Method (ML). Salient loadings (*i.e.*, “significant” associations with a factor) are shown in bold.

**Colorectal cancer tissue**								
	F1*_TU_*		F2*_TU_*		F3*_TU_*		F4*_TU_*	
	PF	ML	PF	ML	PF	ML	PF	ML
*CYP27B1*	**0.73**	**0.61**	0.07	0.15	−0.08	−0.11	0.05	0.15
*VDR*	**0.81**	**0.92**	−0.11	0.01	−0.03	−0.01	0.2	0.19
*COX-2*	**0.34**	**0.38**	**−0.50**	**-0.3**	−0.10	−0.14	−0.02	0.01
subsite	0.2	0.1	**0.62**	**0.9**	−0.12	−0.10	−0.16	−0.13
gender	−0.18	−0.17	**0.33**	0.24	**0.45**	**0.95**	0.18	0.14
age	0	−0.02	−0.12	-0.13	**0.7**	**0.31**	−0.07	0
grading	−0.01	0.04	−0.03	-0.05	0	0.05	**0.4**	0.25
*CYP24A1*	0.24	0.16	−0.03	0.01	0.02	−0.04	**0.5**	**0.77**
**Adjacent Mucosa**								
*CYP27B1*	**1**	**0.99**	0.03	0.08	0.06	0.06	−0.03	0.13
*VDR*	**0.42**	**0.34**	−0.06	−0.04	0.11	0.07	**0.6**	**0.69**
*COX-2*	0.25	0.18	−0.05	−0.03	0.1	0.07	**0.3**	**0.34**
subsite	0.06	0.08	0.16	0.19	−0.25	−0.20	−0.16	-0.15
gender	−0.04	−0.08	**0.77**	**0.63**	0.14	0.18	0.01	0
age	0.12	0.11	0.2	0.15	**0.83**	**0.98**	−0.02	0.02
grading	−0.11	−0.20	0.09	0.1	−0.03	−0.03	**0.52**	**0.49**
*CYP24A1*	**0.32**	0.29	**0.31**	**0.41**	−0.17	−0.14	0.1	0.11

Combined results from principal factor extraction and maximum likelihood methods indicate that Factor 1*_TU_* strongly correlates with expression of *CYP27B1*, *VDR* and *COX-2*. The positive values of all loadings indicate common positive regulation of those three genes. Factor 2*_TU_* displays high positive loadings of tumor subsite as well as salient negative loadings of *COX-2* expression. Loading for gender reached a weight >0.3 by principal factor extraction. Factor 3*_TU_* correlates strongly with age and gender. Factor 4*_TU_* is “significantly” associated with *CYP24A1* expression and grade of malignancy (Table 1).

**Figure 6:**
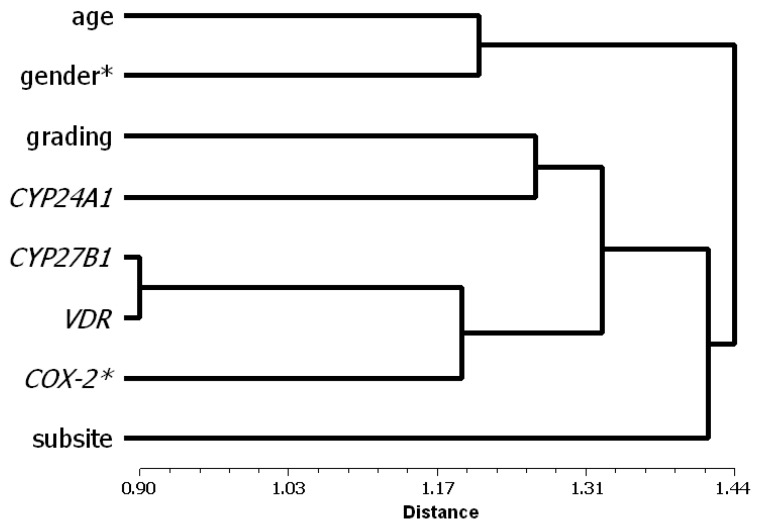
Dendrogram (“hierarchical tree”) showing results of cluster analysis by the UPGMA method with all variables. Asterisk marks variables with salient loadings on two factors in the factor analytical model. On the left of the plot, at the origin of the horizontal axis, each variable is shown as a class by itself.Linkage distance marks the position at which variables can be merged into clusters. Smaller values indicate clusters with strong associations between few variables, whereas clusters at higher values contain more variables though with weaker mutual associations. Patient level analysis of mutual correlations between malignancy, age, gender, subsite location, *CYP27B1*, *CYP24A1*, *VDR* and *COX-2* (see also [2]).

We furthermore assessed the plausibility of the presented factor analytical model by cluster analysis of all variables, since only two out of eight variables (*i.e.*, expression of *COX-2* and gender) had more than one salient loading on factors. The dendrogram shown in Figure 6 reveals a strong confirmation of the presented factor analytical model: Three of the clusters directly correspond to Factors 1*_TU_*, 3*_TU_*, and 4*_TU_*, whereas “subsite” stands alone. Factor 2*_TU_* can be construed as combination of the single branch “subsite” with those variables that display salient loadings not only on the factors represented by their corresponding clusters, *i.e.*, *COX-2* expression and gender. In order to monitor goodness of fit of the resulting dendrogram with the input data, ultrametric distances were calculated from the tree matrix and were correlated with the input average taxonomic distances, yielding a high correlation coefficient of r = 0.855.

When we applied our factor analytical model to the adjacent mucosa, we could also extract four “Factors” though with different loading patterns (Table 1): Factor 1*_AM_* bears salient loadings from *CYP27B1*, *VDR*, and *CYP24A1*, Factor 2*_AM_* from gender and *CYP24A1*, Factor 3*_AM_* from age, and Factor 4*_AM_* from *VDR*, *COX-2* and grading.

## 4. Discussion

Results from mRNA expression analysis (Figure 2) show that transcriptional activities of *CYP27B1*, *CYP24A1* and *COX-2* are significantly elevated in cancerous lesions compared to mucosal tissue outside of the tumor area (*cf.* [21,20]). During tumor progression elevated *CYP27B1* remains constant, whereas *COX-2* peaks in low grade and *CYP24A1* in high grade cancers. As 1,25-(OH)_2_D_3_ induces *CYP24A1* in colon carcinoma cells [7], this could explain, at least in part, the parallel rise of the vitamin D catabolic enzyme during tumor progression to low grade malignancy. However, the extraordinarily high level of *CYP24A1* activity in highly malignant cancers probably does not result from up-regulation by the 1,25-(OH)_2_D_3_/VDR system but may be due to overexpression of the gene. Taken together, expression of the two vitamin D hydroxylases and of *COX-2* relative to each other depends very much on the degree of tumor cell differentiation.

In our cohort we observed a distinct site-specificity, since there is more *COX-2* expression in proximal tumors compared with distal and rectal carcinomas. Sub-group analysis according to gender revealed a significantly higher expression of *CYP27B1*, *VDR* and *COX-2* in the tumors located in the distal colon of male patients compared with that of female patients. In the rectum, only *CYP27B1* showed gender-dependent differences, such that expression was higher in women. *COX-2* showed an even stronger gender-dependency in low-grade tumors with significantly higher expression levels in men compared with women (Figure 3 and Figure 4).

By factor analysis, the eight mutually correlated variables (see Figure 5) could be assigned to four latent, independent variables, so-called factors. These serve as markers for higher categories that cannot be measured directly but represent core features of colorectal cancer pathogenesis. For example, high loadings of *CYP27B1* and *VDR* as well as of *COX-2* on Factor 1*_TU_* (Table 1) indicate that (i) 25-hydroxyvitamin D-1α-hydroxylase-mediated synthesis of 1,25-(OH)_2_D_3_ and its genomic actions via the nuclear VDR are apparently not disrupted in colorectal tissue irrespective of subsite, age, gender, and tumor grading, and (ii) that expression of the 1,25-(OH)_2_D_3_/VDR system and of the inflammation marker *COX-2* are strongly correlated. This can be interpreted as activation of the 1,25-(OH)_2_D_3_/VDR complex in response to inflammatory stimuli in colorectal cancer tissue. Gustafsson Asting *et al*. [22] showed that high *COX-2* expression in colorectal cancer is associated with expression of a host of inflammatory cytokines and mitogenic signaling factors. Particularly the latter have been implied to mediate the up-regulation of the *CYP27B1/VDR* system in early malignancy [5,21]. Taken together, Factor 1*_TU_* stands for *joint activity of the CYP27B1/VDR system and COX-2 in an inflammatory cancerous environment*.

Factor 2*_TU_* captures high *COX-2* expression in proximal colon cancers (Figure 2), which is more pronounced in men than in women. In the analysis, “subsite” was defined as ordinal variable with increasing indices assigned to proximal colon, distal colon, and the rectum, respectively. Likewise, as for the dichotomous variable “gender”, female sex was assigned a higher index than male sex, explaining the negative sign of *COX-2* expression loadings (Table 1). Altogether, this indicates an increase in inflammatory activity in cancerous lesions in the colorectum in the distal to proximal direction, particularly in men. By the same token, the interpretation of an inflammatory component decreasing towards the rectum, particularly in female patients, is equally valid. Factor 2*_TU_* therefore reflects a *subsite- and gender-related inflammatory component* in colorectal cancer development. The proximal colon is certainly the prime site of tumorigenic actions of bile acids. This probably causes a higher incidence of cancers at this site compared to more distal locations in the colorectum. Bile acids such as deoxycholic and lithocholic acid are able to induce pre-neoplastic lesions, so-called aberrant crypt foci, in the colonic epithelium. These are “hot spots” of hyperproliferating pluripotent, undifferentiated cells, which are likely to develop into cancerous lesions (for review, [23]). There is also evidence that bile acids specifically induce *COX-2* expression and, at the same time, suppress expression of 15-hydroxyprostaglandin dehydrogenase, the key enzyme responsible for catabolism of prostaglandins [24]. Notably, some bile acids also interact with the vitamin D system: For example, by binding to the VDR, lithocholic acid stimulates expression of *CYP24A1* [25] and might thus reduce the availability of locally synthesized anti-mitogenic 1,25-(OH)_2_D_3_.

The lower incidence of cancers in the distal parts of the colorectum in women had been linked to direct and indirect anti-proliferative effects of estrogens, which are known to modulate *COX-2* and *CYP27B1/VDR* expression: Thus, 17β-estradiol inhibits prostaglandin E_2_-induced *COX-2* expression [26] and increases *VDR* and *CYP27B1* expression and activity in cultured human colonocytes [27,28,29] as well as in human rectal epithelium *in vivo* [30].

Salient positive loadings of gender and age on Factor 3*_TU_* can be interpreted to account for *gender-specific influence of age* on colorectal cancer pathogenesis. In our cohort, female patients, particularly those with high grade colorectal cancer, were older than their male counterparts (for details, [2]). It has also been reported that younger women with metastatic colorectal cancer survive longer than younger men [31]. The question why women are protected particularly from more aggressive cancer in early life cannot be easily answered. It has been argued that this may be due to long-time exposure to sex hormones before menopause, or during hormone replacement therapy thereafter. It is well established that women on hormone replacement therapy have an approximately 40% lower risk of colorectal cancer, particularly when estrogen therapy is complemented with progestin [32,33]. Because sporadic colon cancers progress stepwise from adenoma to carcinoma, with a latency period that may last decades [34] and with highest incidence during advancing age, it is conceivable that tumors start developing slowly before menopause, but rapidly progress with cessation of ovarian estrogen production.

Factor 4*_TU_* reflects a strong positive correlation between *CYP24A1* and histopathological tumor grading. Factor 4*_TU_* therefore accounts for the relevance of *vitamin D degradation for progression to a higher degree of malignancy.*

The plausibility of the presented factor analytical model was confirmed by cluster analysis of all variables. Cluster analysis aims at sorting different objects into groups in a way that the degree of association between two objects is maximal if they belong to the same group and minimal otherwise. The results can be presented in form of a “hierarchical tree” as for example in Figure 6 (see Legend for details): The cluster that can be formed from *CYP27B1/VDR* and *COX-2* at a short linkage distance, indicating a high degree of association between the variables, corresponds to Factor 1*_TU_* of the factor analytical model. Likewise, the clusters built at a somewhat higher distance from age and gender or from *CYP24A1* and tumor grading reflect Factors 3*_TU_* and 4*_TU_*, respectively. A combination of the single branch “subsite” with a cluster containing the variables with salient loadings on two factors, *i.e.*, *COX-2* and gender, corresponds to Factor 2*_TU_* (Figure 6).

We want to emphasize that Factors 1*_TU_*–4*_TU_* are specific features of malignancy as none of them could be extracted from variables with respect to adjacent mucosal tissue. In fact, Factors 1*_AM_*–3*_AM_* seem to reflect a basically normal situation as they are apparently not influenced by the grade of malignancy of the adjacent tumor: Factor 1*_AM_* indicates activity of the vitamin D endocrine system in normal mucosa. Factor 2*_AM_* denotes a positive association between female gender and *CYP24A1*-encoded 25-(OH)D-24-hydroxylase. Factor 3*_AM_* shows that age is an independent variable in the histologically normal mucosa. Only Factor 4*_AM_* can be seen as indication that progression to higher grades of malignancy makes its imprint on tissue out of the tumor area, as *COX-2* and *VDR* in conjunction with tumor grading became salient (Table 1).

Our results have some important implications for prevention of colorectal cancer. Since inhibition of COX-2-mediated prostaglandin synthesis protects from colorectal cancer only to a certain degree, modulation of colonic vitamin D hydroxylase activities by nutrient factors, as proposed by Cross *et al*. [5,35], should be considered as an additional strategy for prevention. Enhancement of 25-(OH)D-1α-OHase activity will add to availability of 1,25-(OH)_2_D_3_ in the gut epithelium and consequently lessen the tumorigenic potential of COX-2, while simultaneous down-regulation of CYP24A1 could reduce the risk of further tumor progression.

Inhibition of COX-2 cannot be suggested for colorectal cancer prevention in general, because the use of selective as well as non-selective COX-2 inhibitors bears a strong risk of thrombotic cardiovascular events in individuals with a high risk for cardiovascular disease (for review, see [32]). In this case, cancer prevention with vitamin D could turn out as a viable alternative to COX-2 inhibition by non-steroidal anti-inflammatory drugs, because 1,25-(OH)_2_D_3_ has the potential to inhibit COX-2 [33] and to protect from adverse cardiovascular events [36].

## 5. Conclusions

Combined results from factor and cluster analysis suggest that (i) the antagonism between *COX-2* and the anti-proliferative activity of the vitamin D system could be an important determinant of cancer cell growth in the colorectal epithelium; (ii) differences in *COX-2* expression could be, at least in part, responsible for variations of cancer incidence at different anatomical subsites; (iii) gender-specific differences in cancer incidence correlate mainly with advancing age; (iv) progression from well differentiated to undifferentiated cancers correlates with the activity of the vitamin D catabolic enzyme, the *CYP24A1*-encoded 25-hydroxyvitamin D-24-hydroxylase.
